# Contextualizing selection bias in Mendelian randomization: how bad is it likely to be?

**DOI:** 10.1093/ije/dyy202

**Published:** 2018-10-15

**Authors:** Apostolos Gkatzionis, Stephen Burgess

**Affiliations:** 1 MRC Biostatistics Unit, School of Clinical Medicine, University of Cambridge, Cambridge, UK; 2 Cardiovascular Epidemiology Unit, Department of Public Health and Primary Care, School of Clinical Medicine, University of Cambridge, Cambridge, UK

**Keywords:** instrumental variables, causal inference, selection bias, collider bias, inverse probability weighting

## Abstract

**Background:**

Selection bias affects Mendelian randomization investigations when selection into the study sample depends on a collider between the genetic variant and confounders of the risk factor–outcome association. However, the relative importance of selection bias for Mendelian randomization compared with other potential biases is unclear.

**Methods:**

We performed an extensive simulation study to assess the impact of selection bias on a typical Mendelian randomization investigation. We considered inverse probability weighting as a potential method for reducing selection bias. Finally, we investigated whether selection bias may explain a recently reported finding that lipoprotein(a) is not a causal risk factor for cardiovascular mortality in individuals with previous coronary heart disease.

**Results:**

Selection bias had a severe impact on bias and Type 1 error rates in our simulation study, but only when selection effects were large. For moderate effects of the risk factor on selection, bias was generally small and Type 1 error rate inflation was not considerable. Inverse probability weighting ameliorated bias when the selection model was correctly specified, but increased bias when selection bias was moderate and the model was misspecified. In the example of lipoprotein(a), strong genetic associations and strong confounder effects on selection mean the reported null effect on cardiovascular mortality could plausibly be explained by selection bias.

**Conclusions:**

Selection bias can adversely affect Mendelian randomization investigations, but its impact is likely to be less than other biases. Selection bias is substantial when the effects of the risk factor and confounders on selection are particularly large.

**Key Messages**

- In Mendelian randomization experiments, selection bias may arise as a result of collider bias when selection depends on the risk factor and/or the outcome.
- Selection bias is usually small compared with other types of bias if the effects of the risk factor and/or outcome on selection are weak or moderate. However, it can be a real concern if the selection effects are strong.
- Selection bias is increased in the presence of strong confounding. It is also influenced by direct confounder or instrument effects on the selection procedure. It is not affected by instrument strength.
- Inverse probability weighting can be used to adjust for the bias when selection effects are strong and the underlying probability model is correctly specified. However, if selection bias is weak and the probability model is misspecified, inverse probability weighting may even increase the bias.

## Introduction

Mendelian randomization is the use of genetic information to assess the existence of a causal relationship between a risk factor and an outcome of interest.^1^^,^^2^ It is the application of instrumental variable analysis in the context of genetic epidemiology, where genetic variants are used as instruments. To be a valid instrumental variable, a genetic variant must be associated with the risk factor in a specific way—it cannot influence the outcome except via its association with the risk factor, and it cannot be associated with any confounder of the risk factor–outcome association. An association between a valid instrumental variable and outcome is indicative of a causal effect of the risk factor on the outcome.^3^^,^^4^

This paper discusses selection bias in Mendelian randomization. In general, selection bias arises when individuals included in the study population are not a representative sample of the target population.^5^ Selection bias is likely to be present in all epidemiological analyses to some extent. Bias due to non-representative selection usually occurs as an example of collider bias.^6–9^ A collider is a variable that is a common effect of two variables (it is causally downstream of both variables). Collider bias occurs when conditioning on such a variable: even if the two initial variables were unrelated (marginally independent), they will typically become related when conditioning on a collider (conditionally dependent). An example of this is the so-called Berkson’s bias^7^: two diseases A and B that often cause hospitalization may be independent across the population, but they will typically be dependent among hospitalized individuals, since being hospitalized and not having disease A means one is more likely to have disease B.

Throughout this paper, we assume that risk factor–outcome confounding is represented by a single variable, referred to as the confounder. Collider bias in Mendelian randomization studies often results in a violation of the instrumental variable assumptions. By assumption, an instrumental variable and the confounder are marginally independent. Conditioning on a collider of the instrumental variable and the confounder would induce an association between the two^10^ and would lead to the instrumental variable becoming invalid. Hence, selection bias can lead to an association between the instrumental variable and the outcome in the absence of a causal effect of the risk factor on the outcome.^11^

**Figure 1.**
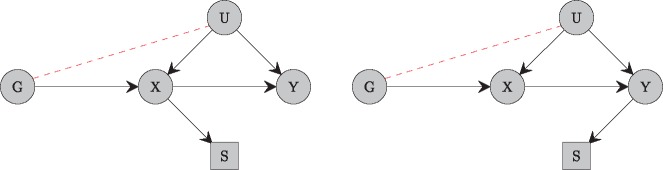
Directed acyclic graphs (DAG) indicating the relationships between an instrumental variable (*G*), exposure (*X*), confounder (*U*) and outcome (*Y*). Selection (*S*) leads to bias if it is a function of the exposure (left panel) or the outcome (right panel), as both exposure and outcome are causally downstream of the genetic variant and confounder, and hence conditioning on selection induces an association between the genetic variant and confounder in both cases.

Collider bias in Mendelian randomization can be visualized through causal diagrams. Directed acyclic graphs indicating the relationships between the genetic variant, risk factor, confounder and outcome are shown in Figure 1. We can see that the risk factor and outcome are both colliders between the genetic variant and the confounder. This means that, if selection into the sample population is a function of the risk factor, then selection bias will occur (Figure 1, left). The same will occur if selection is a function of the outcome (Figure 1, right), but not if it is a function of the confounder alone, as the confounder is not a collider.^12^

The possibility that selection bias may undermine instrumental variable analyses, and Mendelian randomization in particular, has long been noted in the literature.^13^ However, simply saying that selection bias may undermine a Mendelian randomization study is a platitude—it is a true statement, but not a helpful one. Such unhelpful statements are pervasive in epidemiology papers—it is common in the discussion of papers analysing observational data to read bald statements highlighting the possibility that findings could have been adversely affected by selection bias, or similar phenomena such as unmeasured confounding and measurement error. It would be more helpful to evaluate to what extent selection bias is likely to influence findings in terms of bias or Type 1 error rate inflation, or to suggest the magnitude of selection bias that would be required for a positive finding to be explained through bias alone.^14^

In this paper, we aim to contextualize to what extent selection bias affects a typical Mendelian randomization investigation. Our hope is that this paper will help investigators make an informed judgement about the relative importance of selection bias in their work compared with other potential sources of bias. We first list some typical scenarios for Mendelian randomization investigations in which selection bias may occur. We then consider simulated datasets that are similar to applied Mendelian randomization investigations, demonstrating the extent of bias and Type 1 error rate inflation that occurs due to selecting based on a collider. We show how inverse probability weighting can often help reduce bias and re-establish nominal Type 1 error rates, but this sometimes comes at the cost of increased standard errors for the causal effect estimate. The use of weight trimming to avoid this inflation of standard errors is also discussed. The application of inverse probability weighting is illustrated in an example concerning the effect of lipoprotein(a) on coronary heart disease (CHD) risk. We also discuss consequences of inverse probability weighting in terms of the population to which the estimate relates.

## Selection bias in practice

In this section, we provide some examples of Mendelian randomization studies in which selection bias is likely to arise. These are in addition to generic scenarios in which selection bias would occur in any epidemiological analysis, such as the sample population being unrepresentative due to low recruitment rate (e.g. the initial recruitment rate for UK Biobank was 10%^15^) or due to the design of the study (e.g. the Million Veteran Program specifically targets US military veterans^16^).

### Assessing the causal effect of a risk factor on secondary disease or disease progression

Selection bias could occur when considering a secondary disease outcome, such as disease progression. For example, a recent Mendelian randomization investigation considered the effect of body mass index (BMI) on breast-cancer progression.^17^ In order to be included in an analysis of disease progression, a participant must have had an initial disease event. If BMI is a risk factor for breast-cancer risk, then selection into the sample population would be a function of a collider (namely BMI) and hence bias would occur. Bias would also occur for analysis of a recurrent disease event if the risk factor was a cause of the first disease event. For example, lipoprotein(a) [Lp(a)] levels are known to be associated with the risk of CHD. However, a recent study^18^ conducted on individuals with already established CHD reported that Lp(a) was not associated with future cardiovascular mortality; in addition, the two genetic variants in the *LPA* gene region that were used in the study were found not to be associated with cardiovascular mortality. We return to this example later in the paper to assess whether this result could be explained by selection bias.

### Assessing the causal effect of a risk factor on a disease outcome in an elderly population

Another form of selection bias is survivor bias, which may occur when considering a disease outcome in an elderly population.^19^ For example, a recent Mendelian randomization investigation considered the effect of BMI on Parkinson’s disease risk.^20^ Genetic associations with disease risk were estimated in a consortium of studies with mean age varying from 48.9 to 76.2 years. Whereas selection due to mortality may be negligible in a study of young adults, it would be more concerning in an elderly sample population. As above, since BMI is a risk factor for increased mortality, selection bias could occur.

### Assessing a causal effect in a subpopulation

Selection bias could also occur when considering causal effects in a subset of the population. This could result in selection being a function of the risk factor. For example, genetic associations of alcohol-related variants with oesophageal cancer have been considered separately in non-drinkers, moderate drinkers and heavy drinkers^21^—stronger associations would be expected in heavier drinkers. Selection bias would have occurred here, as strata were defined based on the exposure. In contrast, sex-stratified analyses of alcohol-related variants^22^ should not be affected by selection bias, as sex is determined at conception and cannot be an effect (collider) of any other variable.^21^^,^^23^ Alternatively, assessing a causal effect in a subpopulation may result in selection being a function of the outcome. This would be the case if participants were recruited in a hospital setting, and so those with a high risk of the disease are more likely to be selected.

## Simulation study

To investigate the magnitude of bias and Type 1 error inflation in a typical Mendelian randomization investigation, we perform a simulation study. We start with a base scenario, and then vary different parameters of the data-generating model in turn.

### Data-generating model and choice of parameters

Our simulation model is as follows (individuals are indexed by *i*):

$$X_{i}=\alpha_{G}\;G_{i}+\alpha_{U}\;U_{i}+\sqrt{1-\alpha_{G}^{2}-\alpha_{U}^{2}}\;\epsilon_{Xi}$$

$$Y_{i}=\beta_{X}\;X_{i}+\beta_{U}\;U_{i}+\sqrt{1-\beta_{X}^{2}-\beta_{U}^{2}}\;\epsilon_{Yi}$$

$$S_{i}∼\text{Bernoulli}(\pi_{i})$$

$$\text{logit}(\pi_{i})=\gamma_{0}+\gamma_{X}X_{i}+\gamma_{U}U_{i}$$

$$G_{i},U_{i},\epsilon_{Xi},\epsilon_{Yi}∼N(0,1)\;\text{independently}$$

The risk factor (*X*) is a linear combination of the genetic variant (*G*), confounder (*U*) and an independent error term (*ϵ_X_*). The outcome (*Y*) is a linear combination of the risk factor, confounder and an independent error term (*ϵ_Y_*). Selection (*S*) is modelled as a Bernoulli trial with probability of selection *π*, which is assumed to be a logistic-linear function of the risk factor and confounder. The parameter *β_X_* denotes the causal effect to be estimated.

We have specified the model so that the risk factor and the outcome both have standard normal distributions. Consequently, the parameters $\alpha_{G}^{2},\;\alpha_{U}^{2}$ can be interpreted as the proportion of variance in the risk factor that can be explained by the genetic instrument and confounder, respectively, whereas $\beta_{X}^{2}$ and $\beta_{U}^{2}$ have a similar interpretation for the outcome. Finally, the constant term *γ*_0_ determines the prevalence of the selection event (*S *=* *1).

We consider six different simulation scenarios. In the baseline Scenario 1, the parameters are specified as follows. We set $\alpha_{G}=\sqrt{0.02},\;\alpha_{U}=\sqrt{0.5},\;\beta_{U}=\sqrt{0.5}$, meaning that the genetic variant explains 2% of the variance in the risk factor and the confounder explains 50% of the variance in both the risk factor and the outcome. We assume a null causal effect of the risk factor on the outcome ($\beta_{X}=0$). We also set $\gamma_{0}=0$ and $\gamma_{U}=0$, which corresponds to setting the median probability of selection to 0.5 and assuming that the confounder does not influence selection. The risk factor effect on selection *γ_X_* is allowed to take values –2, –1, –0.5, –0.2, 0, 0.2, 0.5, 1 and 2. The odds of selection per 1 standard deviation increase in the risk factor is $\text{exp}(\gamma_{X})$; for $\gamma_{X}=2$, there is a exp(2)=7.39-fold increase in the odds of selection per standard deviation increase in the risk factor.

We then vary in turn: the proportion of variance in the risk factor explained by the genetic variant $\alpha_{G}=\sqrt{0.01},\sqrt{0.05},\sqrt{0.1}$ (Scenario 2); the proportion of variance in the risk factor explained by the confounder $\alpha_{U}=\sqrt{0.2},\sqrt{0.8}$ (Scenario 3); the proportion of variance in the outcome explained by the confounder $\beta_{U}=\sqrt{0.2},\sqrt{0.8}$ (Scenario 4); the effect of the confounder on selection $\gamma_{U}=-1,1$ (Scenario 5); and the probability of selection $\gamma_{0}=-1,-2,-2.4$ (Scenario 6).

We simulate data on a population of 100 000 individuals and then randomly select 10 000 individuals with *S *=* *1 as the sample. In Scenario 6, for $\gamma_{0}=-2$, the analysis is based on a sample size of 1500 instead, as the median probability of selection is 2.3%. For $\gamma_{0}=-2.4$, the analysis is based on a sample size of 500, as the median probability is 0.8%. Ten thousand simulated datasets are generated for each set of parameters. In each simulation, we estimate the causal effect of the risk factor on the outcome using linear regression for the instrument–risk factor and instrument–outcome associations and computing the ratio estimate ${\hat{\beta }}_{X}=\frac{{\hat{\beta }}_{Y|G}}{{\hat{\beta }}_{X|G}}$.

### Results

Results are provided in Table 1 (Scenario 1) and Table 2 (other scenarios). In Table 1, we report the mean, median and standard deviation for the estimated effect of the risk factor on the outcome, the median standard error of these effect estimates and the empirical Type 1 error rate for the risk factor–outcome association at a 5% level of significance level (defined as the proportion of datasets for which $|\frac{{\hat{\beta }}_{X}}{\text{se}({\hat{\beta }}_{X})}|>1.96$). In Table 2, we consider a slightly narrower range of values for the selection effect and only provide the median causal effect estimates and empirical Type 1 error rates.

**Table 1.** Mean, median, standard deviation (SD), median standard error (Med SE) of estimates and empirical Type 1 error rate (%) at a 5% level of significance for associations of the risk factor with the outcome in Scenario 1, for different values of the selection effect (*γ_X_*, also expressed as the odds ratio per 1 standard deviation increase in the risk factor)

*γ_X_*	Odds ratio	Mean	Median	SD	Med SE	Type 1 error rate (%)
–2	0.14	–0.296	–0.289	0.123	0.106	77.7%
–1	0.37	–0.107	–0.103	0.089	0.083	24.3%
–0.5	0.61	–0.032	–0.029	0.077	0.074	6.6%
–0.2	0.82	–0.007	–0.004	0.072	0.071	5.0%
0	1.00	–0.002	0.000	0.071	0.071	5.1%
0.2	1.22	–0.007	–0.004	0.072	0.071	4.8%
0.5	1.65	–0.032	–0.030	0.076	0.074	6.6%
1	2.72	–0.107	–0.103	0.089	0.083	23.6%
2	7.39	–0.296	–0.289	0.123	0.106	77.9%

**Table 2.** Median association of the risk factor with the outcome and empirical Type 1 error rate (%) in Scenario 2 (varying instrument strength), Scenario 3 (varying confounder effect on risk factor), Scenario 4 (varying confounder effect on outcome), Scenario 5 (varying confounder effect on selection probability) and Scenario 6 (varying prevalence of selection) for different values of the selection effect (*γ_X_*)

γ_X_	Median	Type 1 error	Median	Type 1 error	Median	Type 1 error
Scenario 2:	$\alpha_{G}=\sqrt{0.01}$		$\alpha_{G}=\sqrt{0.05}$		$\alpha_{G}=\sqrt{0.1}$	
–1	–0.101	13.9%	–0.104	50.4%	–0.103	79.3%
–0.5	–0.030	5.9%	–0.030	9.8%	–0.029	14.1%
–0.2	–0.004	5.2%	–0.005	5.0%	–0.005	5.3%
0	–0.001	5.0%	–0.001	5.1%	0.000	4.9%
0.2	–0.006	5.3%	–0.005	5.2%	–0.005	5.4%
0.5	–0.027	5.6%	–0.029	9.8%	–0.029	13.8%
1	–0.104	14.0%	–0.103	49.9%	–0.102	79.7%
Scenario 3:	$\alpha_{U}=\sqrt{0.2}$		$\alpha_{U}=\sqrt{0.5}$		$\alpha_{U}=\sqrt{0.8}$	
–1	–0.064	12.1%	–0.105	24.3%	–0.130	35.1%
–0.5	–0.018	5.7%	–0.030	6.6%	–0.039	8.0%
–0.2	–0.003	4.6%	–0.005	5.4%	–0.006	5.1%
0	0.002	4.9%	0.000	4.8%	0.000	5.2%
0.2	–0.004	4.8%	–0.005	5.4%	–0.007	5.1%
0.5	–0.021	5.6%	–0.029	6.6%	–0.038	7.9%
1	–0.067	12.2%	–0.103	24.4%	–0.131	35.8%
Scenario 4:	$\beta_{U}=\sqrt{0.2}$		$\beta_{U}=\sqrt{0.5}$		$\beta_{U}=\sqrt{0.8}$	
–1	–0.065	11.8%	–0.104	24.2%	–0.131	35.5%
–0.5	–0.019	5.7%	–0.029	6.4%	–0.038	7.9%
–0.2	–0.002	5.0%	–0.005	5.1%	–0.007	4.6%
0	0.000	5.3%	–0.001	4.9%	0.000	4.9%
0.2	–0.002	5.1%	–0.003	4.9%	–0.005	5.2%
0.5	–0.018	5.4%	–0.029	6.6%	–0.039	8.0%
1	–0.065	12.1%	–0.100	22.7%	–0.129	34.8%
Scenario 5:	$\gamma_{U}=-1$		$\gamma_{U}=0$		$\gamma_{U}=1$	
–2	–0.293	87.4%	–0.290	78.3%	–0.110	18.1%
–1	–0.145	45.3%	–0.103	24.0%	0.043	8.9%
–0.5	–0.069	16.0%	–0.028	6.9%	0.043	10.0%
–0.2	–0.025	6.6%	–0.004	5.4%	0.023	6.3%
0	0.002	4.9%	0.000	5.0%	–0.001	5.5%
0.2	0.023	6.4%	–0.005	4.8%	–0.025	6.3%
0.5	0.046	9.7%	–0.029	6.4%	–0.068	15.0%
1	0.042	9.1%	–0.101	23.2%	–0.146	45.3%
2	–0.112	18.6%	–0.291	77.7%	–0.293	87.1%
Scenario 6:	$\gamma_{0}=-1$		$\gamma_{0}=-2$		$\gamma_{0}=-2.4$	
–1	–0.103	23.5%	–0.086	6.7%	–0.064	5.4%
–0.5	–0.024	6.4%	–0.019	4.8%	0.000	5.0%
–0.2	–0.007	4.9%	–0.002	5.0%	–0.001	4.9%
0	0.001	4.4%	–0.002	5.2%	–0.006	4.9%
0.2	–0.003	5.2%	0.000	4.9%	–0.002	5.0%
0.5	–0.027	6.3%	–0.018	4.9%	–0.012	5.4%
1	–0.104	24.1%	–0.081	6.9%	–0.072	5.7%

In Scenario 1, when the effect *γ_X_* of the risk factor on selection is weak, the mean causal effect estimates are nearly unbiased. However, as the strength of the selection effect *γ_X_* increases, so does the magnitude of bias. In the rather extreme case where $\gamma_{X}=\pm 2$, bias is so large that the null causal hypothesis is rejected in almost 80% of the simulations.

The direction of selection bias in Table 1 is negative, regardless of the direction or magnitude of the risk factor–selection parameter. The direction of selection bias depends on the confounder effects $\alpha_{U},\beta_{U}$ on the risk factor and the outcome. If *α_U_* and *β_U_* have the same sign, the causal effect estimate is biased downwards; if not, it is biased upwards (Supplementary Table 1, available as Supplementary data at *IJE* online).

In the five scenarios of Table 2, we investigated the impact of varying different parameters on the magnitude of selection bias. In Scenario 2, we varied instrument strength. This did not affect the magnitude of selection bias (see also ^12^). However, stronger instruments led to larger standard errors and hence increased Type 1 error rates. Weak instrument bias is unlikely to have affected our simulations, since we used a single genetic instrument and weak instrument bias is usually small in this case.^24^ Also, even with $\alpha_{G}=\sqrt{0.01}$, the average F statistic for regression of the risk factor on the instrument was around 100.

In Scenarios 3 and 4, we varied the parameters *α_U_* and *β_U_*, representing the confounder effects on the risk factor and the outcome, respectively. In both cases, we observed a moderate increase in the magnitude of selection bias as the strength of the confounder effect increased.

In Scenario 5, we considered a selection procedure influenced by both the risk factor and the confounder. Selection bias is present in this scenario, but the direction of bias also depends on the confounder–selection parameter *γ_U_* in a non-linear and non-monotonic way. In the simulations of Table 2, the causal effect is underestimated if the confounder and risk factor effects on selection have the same direction. It is mildly over-estimated if the risk factor and confounder affect the probability of selection in opposite directions, except when the effect of the risk factor is significantly stronger than that of the confounder, in which case the causal effect is again underestimated. These results also depend on the directions of effects of the confounder on the risk factor and the outcome (Supplementary Table 2, available as Supplementary data at *IJE* online).

Finally, in Scenario 6, there was a weak effect of the probability of selection on selection bias. In particular, bias was slightly reduced when selection was less common. Type 1 error rates also reduced, since simulations for less common selection were based on a smaller sample size, resulting in larger standard errors.

The scenarios that we have considered are by no means exhaustive. Additional scenarios are reported in the Supplementary Material, available as Supplementary data at *IJE* online. When selection depends only on the risk factor, we observed that the magnitude of selection bias is independent of the true value of the risk factor–outcome causal effect (Supplementary Table 3, available as Supplementary data at *IJE* online). When selection is influenced by the outcome only, or by the outcome and confounder, estimates are unbiased when the true causal effect is null (under the causal null, selection is not downstream of the genetic variant and so not a collider; Supplementary Table 4, available as Supplementary data at *IJE* online). However, selection bias is still present when there is a (non-zero) causal effect of the risk factor on the outcome. Finally, when selection depends on the risk factor, selection bias acts similarly in simulations with a binary outcome as with a continuous outcome (Supplementary Table 5, available as Supplementary data at *IJE* online).

In each of the scenarios presented, bias and Type 1 error rate inflation were minimal when $\gamma_{X}=\pm 0.2$ (i.e. each additional standard deviation increase/decrease in the risk factor led to around a 20% greater/lower chance of selection). Bias and Type 1 error rate inflation were minimal, with $\gamma_{X}=\pm 0.5$ (65% greater/40% lower chance per standard deviation increase/decrease in risk factor) in all scenarios except Scenario 2 with $\alpha_{G}=\sqrt{0.1}$ and Scenario 5, in which the confounder also affected selection. Whereas these simulation findings are not universally applicable, in particular the extent of Type 1 error inflation (which would be more severe if the sample size was much bigger or the instrument was much stronger), they suggest that moderate selection bias is unlikely to have a serious impact on moderately sized Mendelian randomization investigations. In comparison, moderate levels of pleiotropy have been shown to lead to more severe bias and Type 1 error inflation.^25^^,^^26^

## Inverse probability weighting

One common solution to the problem of selection bias is to inversely weight the sample population by the probability of selection into the sample.^27^^,^^28^ The intuition is that individuals with low probability of selection are likely to be underrepresented in the sample. Inverse probability weighting upweights these individuals to account for other individuals with similar characteristics in the population who were not included in the sample. For example, if an individual included in the sample population would have been sampled with 100% probability, then that individual does not need to be upweighted whereas, if a selected individual would have been sampled with 20% probability, that individual is effectively replicated four times to represent the 80% of similar individuals who were not sampled.

### Simulations with inverse probability weighting

To investigate the utility of inverse probability weighting to correct for selection bias in Mendelian randomization, we extend the simulations presented in the previous section. We consider Scenario 5 with a varying confounder effect on selection, $\gamma_{U}=-1,0,1$, where $\gamma_{U}=0$ is equivalent to Scenario 1. We perform logistic regression of selection on the risk factor in the full population of 100 000 individuals to estimate the selection probabilities and then perform linear regression of the outcome on the genetic variant weighting by the reciprocals of these probabilities in the 10 000 selected individuals only. For $\gamma_{U}=0$, the selection model is correctly specified whereas, for $\gamma_{U}=\pm 1$, it is not.

### Trimming of weights

A disadvantage of inverse probability weighting is that individuals with a very small probability of selection can receive a very large weight in the analysis. Whereas this is appropriate theoretically, the presence of such individuals can lead to highly variable and imprecise estimates. It is common in practice to trim weights above some threshold^29^—e.g. to set the largest 1% of weights to be equal to the 99th percentile of the empirical distribution of weights. In our simulations, we perform analyses with no trimming, and with trimming at the 99th and 95th percentiles.

### Results

Simulations were repeated for 10 000 datasets for each set of parameters. The results are shown in Table 3. When the inverse probability model was correctly specified ($\gamma_{U}=0$), inverse probability weighting reduced selection bias and the resulting causal effect estimates were unbiased. When the weighting model was not correctly specified ($\gamma_{U}=\pm 1$), bias was present. For small values of *γ_X_*, bias induced by inverse probability weighting was worse than that arising from selection bias. For large values of *γ_X_*, inverse probability weighting did improve bias, even though the weighting model was not correctly specified. In practice, additional information on possible confounders is often available and can also be incorporated in the weighting scheme. Somewhat paradoxically, although increasing the effect of the confounder on the risk factor *α_U_* increases selection bias, it also increases the correlation between the risk factor and confounder, meaning that misspecification in the weighted model based on the risk factor only is less severe (Supplementary Table 6, available as Supplementary data at *IJE* online). Trimming had little effect on results except in the case of extreme values of the risk factor–selection parameter $\gamma_{X}=\pm 2$, where it reintroduced some of the bias that had been removed by using inverse probability weighting, but reduced the variability of estimates.

**Table 3.** Median, standard deviation (SD), median standard error (Med SE) of estimates and empirical Type 1 error rate (%) for the risk factor−outcome causal effect with correctly specified inverse probability weighting selection model ($\gamma_{U}=0$) and misspecified selection model ($\gamma_{U}\pm 1$) for different values of the selection effect (*γ_X_*)

*γ_X_*	Median	SD	Med SE	Type 1	Median	SD	Med SE	Type 1	Median	SD	Med SE	Type 1
$\gamma_{U}=0$	No trimming				Trimming at 99%				Trimming at 95%			
−2	−0.008	6.499	0.072	39.6%	−0.113	0.129	0.085	33.8%	−0.206	0.124	0.096	56.8%
−1	−0.002	0.091	0.071	11.4%	−0.032	0.089	0.075	10.7%	−0.076	0.091	0.080	17.8%
−0.5	−0.002	0.076	0.071	6.3%	−0.010	0.076	0.072	6.3%	−0.027	0.078	0.074	7.2%
−0.2	0.000	0.072	0.071	5.2%	−0.002	0.072	0.071	5.1%	−0.007	0.073	0.072	5.1%
0	0.001	0.072	0.071	5.0%	0.001	0.072	0.071	5.0%	0.001	0.072	0.071	5.0%
0.2	0.001	0.072	0.071	5.0%	−0.001	0.072	0.071	4.9%	−0.006	0.073	0.072	5.1%
0.5	0.001	0.076	0.071	6.5%	−0.008	0.076	0.072	6.4%	−0.024	0.078	0.074	6.7%
1	−0.001	0.091	0.071	11.3%	−0.032	0.089	0.075	10.7%	−0.074	0.092	0.080	17.8%
2	−0.008	0.902	0.072	38.8%	−0.118	0.130	0.085	34.2%	−0.210	0.125	0.096	58.1%
$\gamma_{U}=-1$	No trimming				Trimming at 99%				Trimming at 95%			
−2	−0.031	1.226	0.058	49.0%	−0.130	0.109	0.071	47.3%	−0.207	0.103	0.081	69.5%
−1	0.009	0.110	0.058	24.0%	−0.043	0.086	0.065	17.6%	−0.097	0.086	0.072	30.7%
−0.5	0.025	0.076	0.059	14.7%	−0.003	0.075	0.063	9.5%	−0.040	0.077	0.068	11.6%
−0.2	0.033	0.069	0.061	11.9%	0.016	0.069	0.063	7.9%	−0.010	0.072	0.067	6.7%
0	0.040	0.067	0.063	11.8%	0.029	0.067	0.064	8.8%	0.010	0.069	0.066	6.0%
0.2	0.043	0.066	0.064	10.6%	0.037	0.066	0.065	9.1%	0.024	0.068	0.067	6.8%
0.5	0.049	0.067	0.067	10.9%	0.047	0.067	0.068	10.5%	0.043	0.068	0.068	9.7%
1	0.050	0.074	0.074	10.9%	0.047	0.074	0.074	10.3%	0.041	0.075	0.075	8.9%
2	0.032	0.123	0.086	16.9%	−0.013	0.117	0.093	11.0%	−0.067	0.119	0.100	13.9%
$\gamma_{U}=1$	No trimming				Trimming at 99%				Trimming at 95%			
−2	0.030	0.122	0.087	16.7%	−0.015	0.117	0.093	10.6%	−0.070	0.119	0.100	13.8%
−1	0.052	0.072	0.073	10.9%	0.049	0.072	0.074	10.1%	0.042	0.073	0.075	8.6%
−0.5	0.047	0.067	0.067	11.0%	0.045	0.068	0.067	10.5%	0.041	0.068	0.068	9.5%
−0.2	0.045	0.067	0.064	11.9%	0.039	0.067	0.065	10.0%	0.026	0.069	0.067	7.4%
0	0.039	0.066	0.062	11.4%	0.028	0.067	0.064	8.6%	0.009	0.069	0.066	6.1%
0.2	0.033	0.070	0.061	12.0%	0.016	0.070	0.063	8.1%	−0.011	0.072	0.067	6.9%
0.5	0.025	0.076	0.060	14.1%	−0.004	0.074	0.063	9.1%	−0.042	0.076	0.068	11.5%
1	0.005	0.102	0.058	24.1%	−0.047	0.085	0.065	17.9%	−0.100	0.086	0.072	31.0%
2	−0.034	1.709	0.058	48.5%	−0.132	0.110	0.071	48.0%	−0.209	0.104	0.081	70.2%

## Example: effect of lipoprotein(a) on secondary cardiovascular disease

Lp(a) is an unusual risk factor for Mendelian randomization, as genetic variants in the *LPA* gene region explain up to 90% of its variance.^30^ This comes in contrast to most Mendelian randomization investigations, where genetic variants explain a small proportion of the variance in the risk factor, often as low as 1–5% for sets of genetic variants and polygenic risk scores, and generally less for individual genetic variants. Consequently, even moderate selection bias may have a serious impact on Mendelian randomization analyses of Lp(a). Previous investigations have demonstrated associations between genetic variants in the *LPA* gene region and CHD.^31^^,^^32^ However, a recent investigation of individuals with previous established CHD did not find an association between variants in the same region and subsequent cardiovascular mortality.^18^ We consider by simulation whether this result could be explained by selection bias.

Our data-generating model is the same as in the simulation study except that the outcome is binary:

$$Y_{i}∼\text{Bernoulli}(\pi_{Yi})$$

$$\text{logit}\;\;\pi_{Yi}=\beta_{0}+\beta_{X}X_{i}+\beta_{U}U_{i}.$$

Parameter values are informed by the literature on Lp(a) to resemble the analysis of Zewinger *et al.*,^18^ with the selection variable *S* representing an initial CHD event and the outcome *Y* representing cardiovascular mortality. As in Zewinger *et al.*,^18^ we use a sample size of *n *=* *3313 for the main analysis. This is assumed to be drawn from a larger population of size *N *=* *100 000. We use a single genetic instrument that explains 36% of the variation in Lp(a) levels ($\alpha_{G}=\sqrt{0.36}$); this is the proportion of variation previously reported^31^ as explained by the two variants associated with Lp(a) levels that were used in Zewinger *et al.*’s analysis. We also set $\alpha_{U}=\sqrt{0.32}$, implying that half of the remaining variation in Lp(a) is due to the confounder. We assume that the effect of Lp(a) on CHD risk (the selection event) is equal to the effect of Lp(a) on cardiovascular mortality (the outcome event): $\gamma_{X}=\beta_{X}=+0.25$. Similarly, the effects of the confounder on CHD risk (*γ_U_*) and cardiovascular mortality (*β_U_*) are assumed equal. We set $\gamma_{0}=-2$, meaning that around 20% of the population experience a CHD event and survive to be eligible for selection. We set *β*_0_ to obtain around 20% outcome events in the selected sample (corresponding to the 621 cardiovascular deaths in the original study). We took different values of the confounder effects $\gamma_{U}=\beta_{U}=0,+0.2,+0.5,+1,+1.5,+2$. We generated 10 000 datasets for each value of the confounder effect and calculated in each case the association coefficient from logistic regression for the first 3313 participants in the population (no selection) and the first 3313 with the selection event.

Table 4 shows the results: the mean association estimates with no selection and with selection, and the empirical power under selection. Empirical power for the sample with no selection was not comparable, as there were fewer events in this sample. Even with no selection, the association estimate differed from $\alpha_{G}\times \beta_{X}=0.15$ and attenuated as *γ_U_* increased due to non-collapsibility.^33^^,^^34^ Bias in mean association estimates due to selection was towards the null and increased with *γ_U_*. Although the investigation was well powered in the absence of selection bias, when $\gamma_{U}=+1.5$, bias was fairly severe and the empirical power was 45.5%. When $\gamma_{U}=+2$, the mean association estimate had reduced by almost half compared with the estimate with no selection and empirical power was only 31.0%. Whereas these values of *γ_U_* are fairly large, there are individual cardiovascular risk factors such as LDL-cholesterol that are positively correlated with Lp(a) and do have large effects on CHD risk and cardiovascular mortality; a 30% lowering (approximately 1 standard deviation) of genetically predicted LDL-cholesterol has previously been shown to reduce CHD risk by 67%,^35^ corresponding to $\gamma_{U}=+1.11$ (−log(0.33)=+1.11). When all confounders are considered together, the value of *γ_U_* would be larger still. In conclusion, this simulation exercise suggests that it is plausible that the null finding of Zewinger *et al.* may have been obtained due to selection bias.

**Table 4.** Mean association estimates in the population (‘no selection’) and among individuals with a CHD event (‘with selection’), and empirical power at a 5% level of significance for different magnitudes of confounding in the applied example (the *β*_0_ parameter is chosen such that the proportion of cases in the selected sample is about 20% for each value of *β_U_* and *γ_U_*)

		No selection	With selection	
*β_U_, γ_U_*	β_0_	Mean estimate	Mean estimate	Empirical power
0	–1.4	0.149	0.149	93.5%
+0.2	–1.6	0.148	0.145	91.3%
+0.5	–1.9	0.142	0.133	86.1%
+1	–2.5	0.131	0.102	67.7%
+1.5	–3.3	0.120	0.077	44.0%
+2	–4.0	0.107	0.061	30.4%

## Discussion

The aim of this paper was to consider selection bias in the context of Mendelian randomization. We discussed scenarios in which selection bias may occur, in particular those that are likely to affect Mendelian randomization investigations. We simulated data to be representative of a typical Mendelian randomization investigation and showed that selection bias can significantly influence causal effect estimates when selection into the study is strongly influenced by the risk factor. However, moderate selection bias did not adversely affect estimates too severely across a range of realistic scenarios. A similar conclusion was reached previously for genetic association estimates in the context of secondary events.^36^ Aside from the risk factor–selection parameter, the magnitude of selection bias was shown to be influenced by the strength of the confounder–risk factor and confounder–outcome effects, as well as the confounder–selection parameter and selection frequency. We demonstrated that inverse probability weighting can ameliorate selection bias, but only in cases where the probability of selection can be modelled accurately. When selection bias was moderate, misspecification in the selection model meant that the ‘cure could be worse than the disease’. Finally, we considered a somewhat atypical example of a Mendelian randomization analysis in which genetic variants explained a large proportion of variance in the risk factor, and showed that strong (but credibly so) selection bias could explain the anomalous finding that *LPA* variants were not associated with cardiovascular mortality.

Although inverse probability weighting may be helpful in some cases to reduce selection bias, its implementation requires estimation of probability of selection into the study. This typically requires information on individuals who were not included in the study, which may not be available. An important question when considering whether to use inverse probability weighting is to whom the causal estimate relates. As an example, consider estimating the effect of lipid fractions (in particular, LDL-cholesterol) on cognitive performance after a stroke event. A Mendelian randomization analysis of a representative sample of the general population would provide an estimate of the average causal effect of LDL-cholesterol on cognitive performance in the population as a whole (this may be an average treatment effect or a local average treatment effect, depending on the precise assumptions made^37^—although previous work suggests a Mendelian randomization estimate represents the effect of life-long intervention in a risk factor, and therefore may be a poor guide as to the impact of intervention on the risk factor in a practical setting^38^). Restricting the estimate to those who had a stroke event is likely to lead to selection bias. By inverse probability weighting, we can potentially resolve the problem of selection bias, but now our estimate is reweighted back to the original population—it represents the average effect of intervening on LDL-cholesterol in the population as if everyone in the population had a stroke event. Therefore, by inverse probability weighting, we have resolved the problem that the instrumental variable assumptions were violated in the sample population, but now our causal estimate relates to the general population and not the sample population.

In the majority of simulations in this manuscript, we have modelled selection as depending on the risk factor and a single confounder with linear relationships between variables and the probability of selection as a logistic variable. Although we suspect that our findings will apply to different selection models, it would not be feasible to verify this for every possible model configuration, as well as for binary and time-to-event outcomes. However, our results were robust across a range of realistic scenarios. A potential extension of this work is to develop an analytic bias calculator for instrumental variable analysis. This would be a useful tool for sensitivity analysis not only for Mendelian randomization, but also for other contexts in which instrumental variable analysis is used to analyse observational data.

In conclusion, selection bias can have an adverse effect on Mendelian randomization studies, but in most cases its importance will be less than other sources of bias, such as pleiotropy or population stratification.

## Supplementary Material

dyy202_Supplementary_Material
