# Supplementary material for: Contextualizing selection bias in Mendelian randomization: how bad is it likely to be?
Source: Int J Epidemiol. 2018 Oct 15;48(3):691–701. doi: 10.1093/ije/dyy202 (PMC6659463; doi:10.1093/ije/dyy202)
Supplement: dyy202_Supplementary_Material [file dyy202_supplementary_material.pdf]

Contextualizing selection bias in Mendelian randomization: how bad is it likely to be?

Supplementary Material

Apostolos Gkatzionis [1] and Stephen Burgess [1,2] *

[1] MRC Biostatistics Unit, University of Cambridge, UK

[2] Cardiovascular Epidemiology Unit,
Department of Public Health and Primary Care,
University of Cambridge, UK

July 13, 2018

*Corresponding author: Dr Stephen Burgess. Address: MRC Biostatistics Unit, Cambridge Institute of Public Health, Robinson Way, Cambridge, CB2 0SR, UK. Telephone: +44 1223 748651. Fax: none. Email: sb452@medschl.cam.ac.uk.

Appendix - Additional simulations

We provide additional simulations to further investigate which aspects of a Mendelian randomization study affect the magnitude of selection bias and the performance of inverse probability weighting.

A1 Direction of selection bias

We explored the relationship between the direction of confounder effects on the risk factor and outcome, and the direction of selection bias. For the baseline simulation of Scenario 1, where selection depends only on the risk factor, we varied the signs of these two parameters in our simulations, letting $\alpha_U = \pm\sqrt{0.5}$ and $\beta_U = \pm\sqrt{0.5}$. Results are reported in Supplementary Table A1. The simulation results indicate that the causal effect is biased downwards if the directions of the confounder effects on the risk factor and the outcome are the same, and upwards otherwise.

[Supplementary Table A1]

Note that we have made the simplifying assumption that the confounder U represents the cumulative effect of all possible sources of confounding for the risk factor–outcome association, so α_U and β_U represent the total effect of all confounders on the risk factor and the outcome. In practice, the signs of these parameters may be difficult to determine if different confounders have opposite effects on the risk factor or the outcome.

We also performed additional simulations, summarized in Supplementary Table A2, to assess the direction of selection bias when selection depends on both the risk factor and the confounder. For simplicity, we focus only on the direction of bias and ignore its magnitude.

[Supplementary Table A2]

A change in the direction of bias (a "±" or a "∓" sign) is observed when the signs of γ_U and $\gamma_X \alpha_U$ are different. Intuitively, these parameters express the direct (γ_U) and indirect ($\gamma_X \alpha_U$, mediated by the risk factor) effect of the confounder on selection. If these two effects act on the same direction, the direction of selection bias is determined again by the effects α_U, β_U of the confounder on the risk factor and the outcome, as in Supplementary Table A1. When γ_U and $\gamma_X \alpha_U$ have opposite signs, the confounder affects selection in two opposite ways. Selection bias due to the confounder effect as mediated by the risk factor acts in the direction dictated by the α_U, β_U coefficients, as discussed previously, while selection bias due to the direct effect of the confounder on selection acts in the opposite direction. The relative magnitudes of $\gamma_X \alpha_U$ and γ_U determine which effect is stronger, and hence the direction of bias. In the simulations in the main body of the paper (Scenario 5), γ_X was the only parameter whose value we varied, so the direction of bias depended on that parameter.

A2 Selection bias for a non-null causal effect

To investigate whether selection bias depends on the true value of the risk factor-outcome causal effect, we reproduced the simulations of Table 1 with the causal effect parameter set to $\beta_X = 0.5$ instead of $\beta_X = 0$. Supplementary Table A3 contains the results of this simulation. The magnitude of selection bias was very similar to that reported in Table 1. This implies that when selection only depends on the risk factor, the magnitude of selection bias is independent of the value of the causal effect β_X. Similar results (not reported here) were obtained for a range of different β_X values, as well as for a negative causal effect ($\beta_X = -0.5$).

[Supplementary Table A3]

A3 Outcome-dependent selection mechanism

The selection mechanism used in the simulations of Tables 1 and 2 depended only on the risk factor, except in Scenario 5 where selection also depended on the confounder. Here, we consider an alternative selection procedure, in which selection depends on the outcome and possibly on the confounder (see Figure 1.b of the main document). Applications in which selection depends on the outcome are not uncommon in practice. For example, consider an analysis studying a disease outcome, where data are collected from hospital admission registries. Selection bias on the outcome will occur, since hospitalized individuals are more likely to suffer from the disease studied. Survivor bias can also arise as a result of selection on the outcome, for example if a study samples individuals at random from an elderly population and the outcome studied is all-cause mortality or relates to a life-threatening disease such as cancer.

To implement the simulations, we modified the data-generating model by letting the probability of selection depend on the outcome and the confounder:

$$\text{logit}(\pi_i) = \gamma_0 + \gamma_U U_i + \gamma_Y Y_i.$$

Simulations were performed by varying the strength of the outcome–selection parameter γ_Y, allowing it to take values $-2, -1, -0.5, -0.2, 0, 0.2, 0.5, 1, 2$, and the confounder–selection parameter γ_U, allowing it to take values 0 and +1. All other parameters were the same as in Scenario 1.

As illustrated in Supplementary Table A4, there is no selection bias under the null causal hypothesis ($\beta_X = 0$). Additionally in this case, nominal Type 1 error rates are maintained. Therefore a Mendelian randomization study in which selection depends only on the outcome (and possibly on the confounder) will not lead to false positive results. It is possible that a null finding may be a false negative result due to selection bias, but it is somewhat less likely – it would only occur if selection bias was of the same magnitude as the causal effect and acted in the opposite direction.

On the other hand, when the causal effect parameter β_X is non-zero, causal effect estimates exhibit noticeable bias for strong selection effects. The simulation results of Table A4 illustrate that the direction of selection bias is the same as in simulations with selection on

exposure. The magnitude of bias is slighly reduced compared to the selection-on-exposure simulations (Table 1) and the corresponding standard errors are also lower.

[Supplementary Table A4]

A4 Binary outcomes

So far in our simulations, we have focused on quantifying the effect of selection bias in Mendelian randomization studies with a continuous outcome variable. Studying a binary outcome (such as disease status) is also common in practice, so we briefly investigate this case here. We note that in the context of genetic association studies, a few authors have already suggested that the impact of selection bias may only be modest when a binary outcome is studied (see [9] and references therein).

We performed a set of simulations using a logistic-linear model to simulate the binary outcome, as in the lipoprotein(a) application. In this case, the causal effect represents the log odds ratio for the outcome per unit increase in the risk factor. In our simulations, we set the causal effect equal to $\beta_X = 0$ and let the remaining parameters take the same values as in Scenario 1. We then varied the constant term β_0, which dictates the prevalence of the disease outcome in the population. We allowed β_0 to take values 0, -1.4 and -3, corresponding approximately to an average disease prevalence of 50%, 20% and 5% respectively.

[Supplementary Table A5]

Results are reported in Supplementary Table A5. Selection bias is present here, and the magnitude of bias is similar to that for a continuous outcome. The disease prevalence parameter β_0 has practically no effect on the magnitude of selection bias, but a rare disease (small β_0) is associated with an increased standard error for the causal effect estimate. In general, the standard error will be minimized when disease frequency is about 50%, as happens in a case-control setting.

It is perhaps worth discussing case-control studies in more detail, since they are a common example of epidemiological studies with a binary outcome and Mendelian randomization is sometimes performed on case-control data. In principle, selection into a case-control study depends on the outcome; however, this dependence will not necessarily introduce bias. In a well-designed case-control study, the cases will constitute a random subsample of the population of cases (or even the entire population, if data is available) and the controls will be a random subsample of the population of controls. The only atypical aspect of such a sample compared to the overall population is the frequency of cases, and this is not enough to cause selection bias as Supplementary Table A5 illustrates. Similar findings have been reported outside the context of Mendelian randomization (for example, [9]).

Finally, additional simulations (not reported here) suggested that the performance of inverse probability weighting with a binary outcome is similar to that with a continuous outcome.

A5 Inverse probability weighting with a misspecified weighting model

Inverse probability weighting can yield biased estimates if the model for computing the weights is misspecified. Nevertheless, for the simulations in this paper, selection depended on the risk factor and the confounder but a reasonable approximation to the true causal effect was obtained via weighting by the risk factor only.

The behaviour of inverse probability weighting can be significantly worse if the confounder only has a weak influence on the risk factor. We illustrate this by conducting a simulation similar to that of Table 3, with a weak confounder effect on the risk factor. We set $\gamma_U = 1$ and $\alpha_U = \sqrt{0.1}$ and leave the other parameters unchanged. Results are presented in Supplementary Table A6.

In this simulation, causal effect estimates are subject to significant bias when the risk factor–selection effect is strong. This is the case even when using the inverse probability weighting approach. Again, trimming weights was of little consequence in this example.

[Supplementary Table A6]

Simulation Tables

γ_X	$\alpha_U = -\sqrt{0.5}$, $\beta_U = \sqrt{0.5}$ Median	SD	Med SE	Type 1	$\alpha_U = \sqrt{0.5}$, $\beta_U = -\sqrt{0.5}$ Median	SD	Med SE	Type 1	$\alpha_U = -\sqrt{0.5}$, $\beta_U = -\sqrt{0.5}$ Median	SD	Med SE	Type 1
-2	0.290	0.122	0.106	78.1 %	0.292	0.119	0.106	78.3 %	-0.289	0.121	0.106	77.7 %
-1	0.103	0.089	0.083	23.4 %	0.102	0.089	0.083	23.0 %	-0.104	0.089	0.083	24.1 %
-0.5	0.029	0.076	0.074	7.0 %	0.031	0.076	0.074	6.6 %	-0.029	0.077	0.074	7.1 %
-0.2	0.004	0.071	0.071	4.6 %	0.005	0.072	0.071	5.2 %	-0.005	0.072	0.071	5.1 %
0	0.000	0.070	0.071	4.8 %	-0.001	0.072	0.071	5.1 %	-0.001	0.071	0.071	5.0 %
0.2	0.006	0.072	0.071	5.0 %	0.005	0.073	0.071	5.3 %	-0.005	0.072	0.071	5.1 %
0.5	0.029	0.077	0.074	6.7 %	0.029	0.077	0.074	6.9 %	-0.028	0.075	0.074	6.6 %
1	0.102	0.089	0.083	23.4 %	0.103	0.087	0.083	23.0 %	-0.102	0.089	0.083	23.2 %
2	0.292	0.120	0.106	78.7 %	0.288	0.122	0.106	77.4 %	-0.289	0.121	0.106	77.9 %

Supplementary Table A1: Median, standard deviation (SD), median standard error and 5% empirical Type 1 error rate of- causal effect estimates, for varying directions of the confounder-exposure (α_U) and the confounder-outcome (β_U) effects.

		$\alpha_U > 0$		$\alpha_U < 0$	
		$\beta_U > 0$	$\beta_U < 0$	$\beta_U > 0$	$\beta_U < 0$
$\gamma_U > 0$	$\gamma_X > 0$	$-$	$+$	$\mp$	$\pm$
	$\gamma_X < 0$	$\pm$	$\mp$	$+$	$-$
$\gamma_U < 0$	$\gamma_X > 0$	$\pm$	$\mp$	$+$	$-$
	$\gamma_X < 0$	$-$	$+$	$\mp$	$\pm$

Supplementary Table A2: Direction of selection bias of causal effect estimates when selection depends on the risk factor and the confounder. "+": upward bias, "−": downward bias, "±": upward bias for moderate X-S associations, downward bias for strong associations, "∓": downward bias for moderate X-S associations, upward bias for strong associations.

γ_X	Odds ratio	Mean	Median	SD	Med SE	Empirical Power
-2	0.14	0.203	0.211	0.108	0.118	42.6 %
-1	0.37	0.392	0.396	0.078	0.098	98.1 %
-0.5	0.61	0.466	0.468	0.066	0.090	99.9 %
-0.2	0.82	0.492	0.494	0.061	0.087	100.0 %
0	1.00	0.498	0.500	0.062	0.086	100.0 %
0.2	1.22	0.493	0.495	0.063	0.087	100.0 %
0.5	1.65	0.468	0.471	0.066	0.090	100.0 %
1	2.72	0.392	0.397	0.078	0.098	98.0 %
2	7.39	0.205	0.211	0.108	0.119	43.2 %

Supplementary Table A3: Mean, median, standard deviation (SD), median standard error and empirical power to reject the null causal hypothesis at a 5% significance level for causal effect estimates in Scenario 1, with the true causal effect set to $\beta_X = 0.5$.

γ_Y	$\beta_X = 0$				$\beta_X = 0.5$			
$\gamma_U = 0$	Median	SD	Med SE	Type 1 Error	Median	SD	Med SE	Emp Power
-2	0.000	0.057	0.056	5.2 %	0.336	0.063	0.076	99.2 %
-1	0.001	0.066	0.064	5.5 %	0.420	0.062	0.082	100.0 %
-0.5	0.001	0.070	0.069	5.1 %	0.474	0.061	0.085	100.0 %
-0.2	0.000	0.071	0.070	5.1 %	0.495	0.061	0.086	100.0 %
0	-0.001	0.070	0.071	4.5 %	0.500	0.062	0.086	100.0 %
0.2	0.000	0.071	0.070	5.1 %	0.496	0.062	0.086	100.0 %
0.5	0.000	0.069	0.069	5.2 %	0.474	0.063	0.085	100.0 %
1	0.000	0.065	0.064	5.3 %	0.419	0.063	0.082	99.9 %
2	0.001	0.057	0.056	5.2 %	0.335	0.061	0.076	99.3 %
$\gamma_U = 1$	Median	SD	Med SE	Type 1 Error	Median	SD	Med SE	Emp Power
-2	-0.001	0.064	0.063	5.2 %	0.328	0.069	0.086	96.8 %
-1	-0.001	0.071	0.070	4.9 %	0.468	0.064	0.088	99.9 %
-0.5	0.000	0.071	0.070	5.2 %	0.512	0.060	0.085	100.0 %
-0.2	0.000	0.069	0.069	4.9 %	0.509	0.059	0.082	100.0 %
0	0.000	0.067	0.068	4.4 %	0.500	0.058	0.081	100.0 %
0.2	0.000	0.067	0.066	4.9 %	0.486	0.059	0.079	100.0 %
0.5	-0.001	0.064	0.064	4.9 %	0.462	0.057	0.077	100.0 %
1	0.000	0.060	0.059	4.8 %	0.423	0.057	0.074	100.0 %
2	-0.001	0.055	0.053	5.6 %	0.364	0.056	0.070	100.0 %

Supplementary Table A4: Median, standard deviation (SD), median standard error and empirical power to reject the null causal hypothesis at a 5% significance level (for $\beta_X = 0$, this is equal to the empirical Type 1 error rate) for causal effect estimates where selection depends only on the outcome ($\gamma_U = 0$) or on the outcome and the confounder ($\gamma_U = 1$).

γ_X	$\beta_0 = 0$				$\beta_0 = -1.4$				$\beta_0 = -3$			
	Median	SD	Med SE	Type 1	Median	SD	Med SE	Type 1	Median	SD	Med SE	Type 1
-2	-0.269	0.233	0.225	22.1 %	-0.279	0.305	0.295	15.9 %	-0.301	0.570	0.553	8.7 %
-1	-0.093	0.173	0.171	8.5 %	-0.102	0.223	0.219	7.7 %	-0.106	0.408	0.402	5.9 %
-0.5	-0.027	0.151	0.150	4.9 %	-0.030	0.189	0.187	5.4 %	-0.027	0.341	0.339	5.0 %
-0.2	-0.006	0.144	0.143	5.2 %	-0.009	0.177	0.175	5.2 %	-0.008	0.318	0.313	5.2 %
0	-0.002	0.143	0.141	5.2 %	0.000	0.172	0.171	4.9 %	0.001	0.301	0.302	4.9 %
0.2	-0.006	0.145	0.143	5.2 %	0.000	0.174	0.170	5.3 %	-0.001	0.304	0.299	5.2 %
0.5	-0.027	0.153	0.150	5.6 %	-0.024	0.178	0.176	4.9 %	-0.021	0.307	0.305	5.0 %
1	-0.095	0.174	0.171	8.2 %	-0.093	0.199	0.196	7.8 %	-0.100	0.343	0.336	6.4 %
2	-0.273	0.235	0.225	22.4 %	-0.260	0.256	0.251	17.4 %	-0.277	0.431	0.424	9.8 %

Supplementary Table A5: Median, standard deviation (SD), median standard error and 5% empirical Type 1 error rate for risk factor-outcome causal effect estimates, in simulations with a binary outcome and a varying outcome frequency (50%, 20% and 5%, for $\beta_0 = 0, -1.4, -3$ respectively) for different values of the selection effect (γ_X).

$\gamma_U = 1$	Med	SD	Med SE	Type 1	Med	SD	Med SE	Type 1	Med	SD	Med SE	Type 1
γ_X	No trimming				Trimming at 99%				Trimming at 95%			
-2	0.158	0.112	0.080	51.1 %	0.134	0.105	0.088	37.3 %	0.108	0.106	0.097	22.9 %
-1	0.101	0.074	0.072	29.3 %	0.096	0.075	0.074	26.2 %	0.088	0.078	0.076	21.7 %
-0.5	0.053	0.069	0.069	12.5 %	0.053	0.069	0.069	12.2 %	0.051	0.070	0.070	11.8 %
-0.2	0.024	0.068	0.068	6.7 %	0.024	0.068	0.068	6.7 %	0.023	0.068	0.068	6.5 %
0	0.003	0.067	0.067	5.3 %	0.002	0.068	0.067	5.2 %	-0.001	0.069	0.068	5.1 %
0.2	-0.016	0.068	0.065	6.6 %	-0.019	0.069	0.066	6.8 %	-0.024	0.071	0.068	7.3 %
0.5	-0.045	0.071	0.065	13.0 %	-0.050	0.072	0.067	13.9 %	-0.060	0.075	0.070	15.3 %
1	-0.086	0.080	0.064	31.4 %	-0.101	0.080	0.068	33.6 %	-0.118	0.083	0.074	36.0 %
2	-0.158	1.325	0.066	61.3 %	-0.192	0.107	0.077	65.5 %	-0.220	0.105	0.088	68.2 %

Supplementary Table A6: Median, standard deviation (SD), median standard error (med SE) of estimates and empirical Type 1 error rate (%) for risk factor-outcome causal associations with a misspecified inverse probability weighting model ($\gamma_U = 1$) and a weak confounder–risk factor effect ($\alpha_U = \sqrt{0.1}$), for different values of the selection effect (γ_X).
